# Supplementary material for: PgMYB2, a MeJA-Responsive Transcription Factor, Positively Regulates the Dammarenediol Synthase Gene Expression in Panax Ginseng
Source: Int J Mol Sci. 2019 May 6;20(9):2219. doi: 10.3390/ijms20092219 (PMC6539309; doi:10.3390/ijms20092219)
Supplement: Supplementary file 1 [file ijms-20-02219-s001.pdf]

International Journal of *Molecular Sciences*

MDPI

Article

PgMYB2, a MeJA-Responsive Transcription Factor, Positively Regulates the Dammarenediol Synthase Gene Expression in *Panax ginseng*

Tuo Liu [1,†], Tiao Luo [1,2,†], Xiangqian Guo [1], Xian Zou [1], Donghua Zhou [2], Sadia Afrin [1], Gui Li [1], Yue Zhang [1], Ru Zhang [3], and Zhiyong Luo [1,*]

1 Department of Biochemistry and Molecular Biology, School of Life Sciences , Central South University, Changsha 410008, China; lt1994@csu.edu.cn (Tu.L.); tiaooul96@163.com (Ti.L.); gxq199x@163.com (X.G.); zx13618463547@163.com (X.Z.); nilabotdu@yahoo.com (S.A.); ligui20061029@126.com (G.L.); zhang1045242781@126.com (Y.Z.)

2 School of Stomatology of Changsha Medical University, Changsha 410006, China; csyxyzdh@163.com

3 College of Chemistry and Chemical Engineering, Hunan Institute of Engineering, Xiangtan 411104, China; zhangru2002@126.com

* Correspondence: luozhiyong@csu.edu.cn; Tel.: +86-731-8480-5025

† These authors contributed equally to this work.

Supplementary Materials:

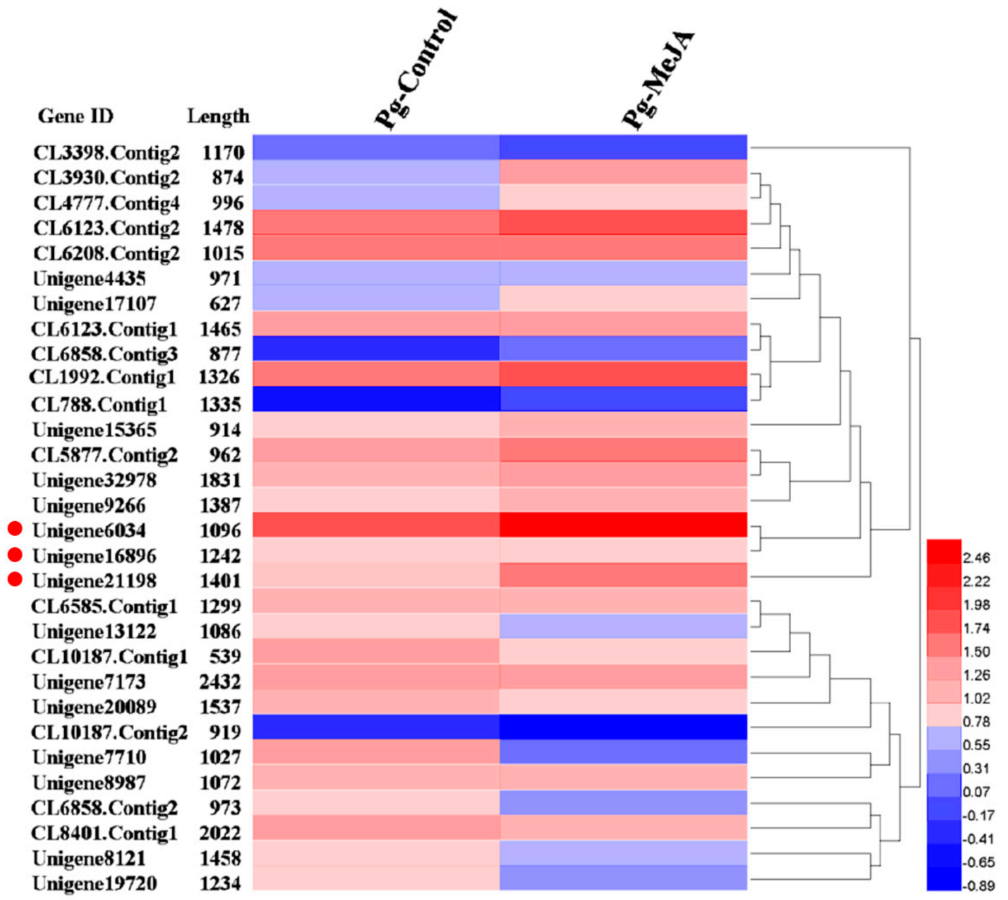

Figure S1. Heatmap of 30 *R2R3-MYB* unigenes. The levels of expression were determined based on comparison of the intensity of two colors (red and blue) from two samples (Pg-Con and Pg-MeJA). The gradation of color indicated different values which were measured using lg (FPKM). The values

were shown with the 'color-scale' at the right-bottom. The red circles represented three unigenes for subsequent screening. The heatmap was generated by HemI program (http://hemi.biocuckoo.org/index.php).

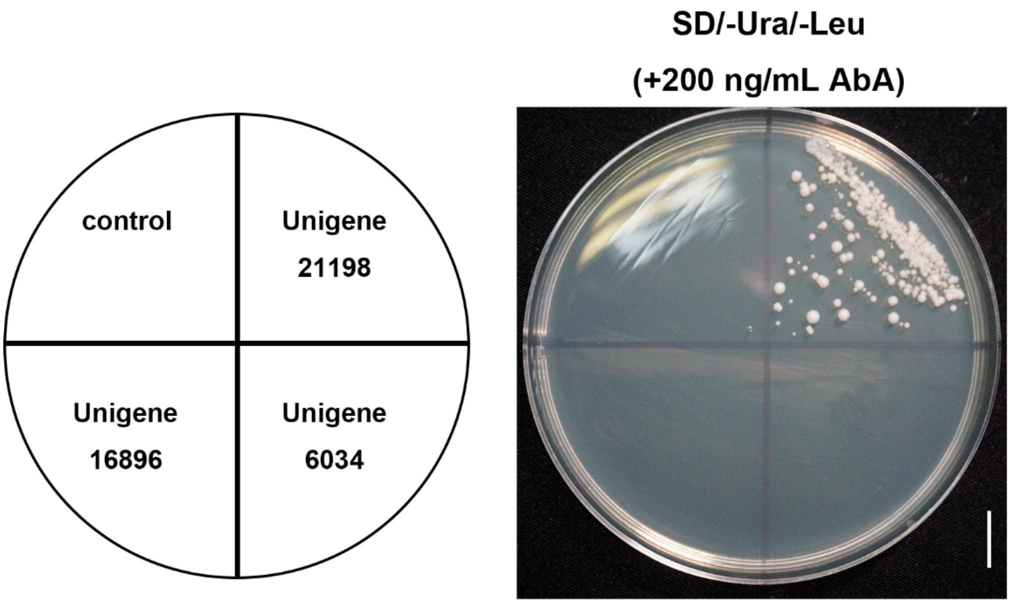

Figure S2. The screening assay of 3 unigenes in Y1H Gold. The pABAi-*DDSpro* were used as the bait and the pGADT7-Rec used as the control. Only the yeast cells with pGADT7-Unigene 21198 (*PgMYB2)* could grow on the SD/-Ura/-Leu selective medium added with 200 ng/mL AbA. Scale bar = 1 cm.

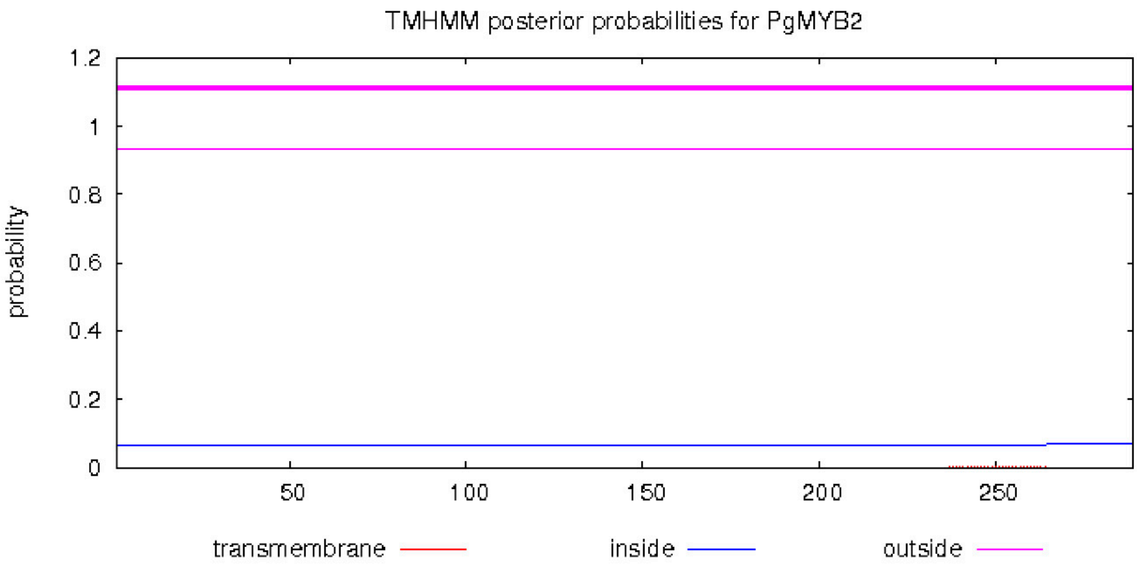

Figure S3. The transmembrane domain of PgMYB2. Predicted by TMHMM Server v. 2.0 (http://www.cbs.dtu.dk/services/TMHMM/).

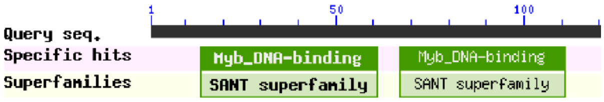

Figure S4. The MYB DNA binding sites of PgMYB2. Predicted by the NCBI conserved domains finder (https://www.ncbi.nlm.nih.gov/Structure/cdd/wrpsb.cgi).

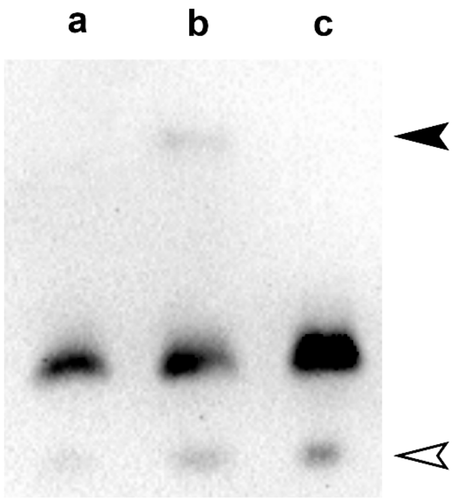

Figure S5. Binding assay of PgMYB2 to *MBS* site. Contents of reaction: (**a**) Free probe; (**b**) labeled probe (containing *MBSII* site) + PgMYB2: TF protein; (**c**) labeled probe (containing *MBS* site) + PgMYB2: TF protein. The protein-probe complexes were indicated with a solid arrow and the free probes were indicated with a hollow arrow.

Table S1. Specific primers used in the study.

**Primer Name**	**Primer sequence (5′ to 3′)**
PgMYB2-F	CCACTCTCAACGCATTCTC
PgMYB2-R	TAGGGGTAGGCACATTCTG
PgDDS-F	ATGTGGAAGCAGAAGGTT
PgDDS-R	AATTTTGAGCTGCTGGTG
β-actin-F	TGCCCCAGAAGAGCACCCTGT
β-actin-R	AGCATACAGGGAAAGATCGGCTTGA
1302-PgMYB2-F	AGAACACGGGGGACTCTTGACCAAGAAGAAATTGACGACGATG
1302-PgMYB2-R	GTGAAAAGTTCTTCTCCTTTACTATTCCTTTTCCCAACAGTCC
qPCR-*PgMYB2*-F	CGGATTATTGAGATGCGG
qPCR-*PgMYB2*-R	TGATGTGGGTGTTCCAGTAGTTC
qPCR-*PgDDS*-F	TGAGATTAGATGAAAACGAAC
qPCR-*PgDDS*-R	GGCAATGATAAGGGGAGGTGT
pABAi-*DDSpro*-F	AATTCGAGCTCGGTACCCGGGCTTGTAGTTTTGTGATTTTCC
pABAi-*DDSpro*-R	ATACAGAGCACATGCCTCGAGACTTGTTGGTATGTGGTGTAA
pGADT7-*PgMYB2*-F	CATATGGCCATGGAGGCCAGTATGATGGGACGTTCACCTTGC
pGADT7-*PgMYB2*-R	ATCTGCAGCTCGAGCTCGATGTCTATTCCTTTTCCCAACAGTCC
pCold/TF-*PgMYB2*-F	ATGGAGCTCGGTACCCTCGAGATGGGACGTTCACCTTGC
pCold/TF-*PgMYB2*-R	AGACTGCAGGTCGACAAGCTTATGTTTTCCCAACAGATGA
EMSA-*MBSI*-F	ACACGTCTAACACGTCAATTCTTTT
EMSA-*MBSI*-R	AAAAGAATTGACGTGTTAGACGTGT
EMSA-*MBSII*-F	GACTGGCATTGATTTAAAAGGCGGT
EMSA-*MBSII*-R	ACCGCCTTTTAAATCAATGCCAGTC
EMSA-mutant-*MBSII*-F	GACTGGCttccgggcacctGGCGGT
EMSA-mutant-*MBSII*-R	ACCGCCaggtgcccggaaGCCAGTC
pGreenII 0800-*DDSpro*-F	GACTAGTTTCTTCCAATACTTGTAG
pGreenII 0800-*DDSpro*-R	CATGCCATGGCATTCTTAAGTCTACTAC
pEGAD-*MYC-PgMYB2*-F	CCGGAATTCATGGGACGTTCACCTTGC
pEGAD-*MYC-PgMYB2*-R	CGCGGATCCACAATATCTGTAAAACCCA

pGreenII 0800-*DDSpro-xMBSII*-F	AAATCTGTCTCTGCTGACAAAAATTCCTAGACC
pGreenII 0800-*DDSpro-xMBSII*-R	GTCAGCAGAGACAGATTTTTGTTAAAGTTAAATAAAGAAAACTTGAACTA

MBS, MBSII and its mutation sites are underlined in the table. These primers were designed by Premier 5, Oligo 7 and SnapGene software.
